# Anxiety, Depression, and Body Weight in Children and Adolescents With Migraine

**DOI:** 10.3389/fpsyg.2020.530911

**Published:** 2020-10-28

**Authors:** Samuela Tarantino, Laura Papetti, Alessandra Di Stefano, Valeria Messina, Fabiana Ursitti, Michela Ada Noris Ferilli, Giorgia Sforza, Romina Moavero, Federico Vigevano, Simonetta Gentile, Massimiliano Valeriani

**Affiliations:** ^1^Unit of Clinical Psychology, Department of Neuroscience and Neurorehabilitation, Ospedale Pediatrico Bambino Gesù, Istituto di Ricovero e Cura a Carattere Scientifico (IRCCS), Rome, Italy; ^2^Headache Center, Division of Neurology, Ospedale Pediatrico Bambino Gesù, Istituto di Ricovero e Cura a Carattere Scientifico (IRCCS), Rome, Italy; ^3^Child Neurology and Psychiatry Unit, Tor Vergata University of Rome, Rome, Italy; ^4^Center for Sensory-Motor Interaction, Aalborg University, Aalborg, Denmark

**Keywords:** children, migraine frequency, body weight, anxiety, depression

## Abstract

**Background:** There is a lack of studies that explore the possible association between body weight, psychological symptoms, and migraine severity in pediatric populations. The purpose of the study was to explore: (1) the association between body weight and the frequency of migraine attacks, (2) the possible differences in anxiety and depression symptoms according to the frequency of attacks and body weight, and (3) the possible mediating role of anxiety and/or depression in the association between body weight and frequency of migraine attacks in children.

**Methods:** One hundred and eleven children/adolescents with migraine were included (47 boys and 64 girls; mean age 11.7; ±2.4 years). The patients were classified as: (1) high frequency patients, reporting from weekly to daily episodes and (2) low frequency patients, with ≤3 episodes per month. According to their body mass index percentiles, the patients were divided in “Normal weight” (from ≥5 to <85 percentile), “Overweight” (from ≥85 to <95 percentile), and “Obese” (≥95 percentile). Given the low number of obese patients, the overweight and obese groups were considered together in the “Overweight” group. Anxiety and depression symptoms were assessed by the Self-Administered Psychiatric Scales for Children and Adolescents (SAFA).

**Results:** Fifty-four patients were normal in weight (49.6%), while 56 patients (50.4%) were overweight. The overweight patients showed a higher frequency of migraine attacks (64.7%; *p* < 0.05). Patients with a high frequency of attacks reported higher scores in all SAFA-Anxiety subscales (SAFA-A Tot: *F* = 15.107; *p* = 0.000). Overweight patients showed a significantly higher score in the “Separation anxiety” subscale (*F* = 7.855; *p* = 0.006). We found a mediating role between the overweight and high frequency for total anxiety (*z* = 2.11 ± 0.03; *p* < 0.05) and social anxiety (*z* = 2.04 ± 0.03; *p* < 0.05).

**Conclusions:** Our results suggest that, among the children suffering from migraine, the overweight status is associated with a higher frequency of attacks and separation anxiety symptoms. In particular, our study provides the first evidence of the role of anxiety in linking overweight and the frequency of migraine attacks in children and adolescents.

## Introduction

Migraine and obesity are very common problems in the pediatric age (Verrotti et al., 2012; Oakley et al., 2014; Farello et al., 2017). Previous epidemiological studies showed a prevalence of migraine ranging from 3% in preschool age to 8–23% during adolescence (Bellini et al., 2013; Genizi et al., 2016). Similarly, the pediatric overweight and obesity are more prevalent over the last decades (Verrotti et al., 2012; Farello et al., 2017). Both conditions are multifactorial diseases, influenced by genetic, environmental, and psychological factors (Strauss, 2000; Lipton and Bigal, 2005; Friedman and De ver Dye, 2009; Albuquerque et al., 2017; Marmura, 2018), are associated with a poor quality of life, high healthcare costs, and increased risk of psychological disorders (Abu Bakar et al., 2016; Apovian, 2016; Öztop et al., 2016). In particular, patients suffering from high frequency of migraine attacks are more likely to report a more negative life impact due to pain (Malone et al., 2015).

In recent years, a growing body of literature analyzed the association between obesity and migraine, both in adult (Bigal et al., 2006; Bigal and Lipton, 2006; Winter et al., 2012; Chai et al., 2014a,b; Ornello et al., 2015; Togha et al., 2019) and pediatric ages (Verrotti et al., 2012; Oakley et al., 2014; Ravid, 2014; Eidlitz-Markus et al., 2015; Farello et al., 2017). Many studies supported the role of being overweight on headache severity and, in particular, in the transformation of migraine from episodic to chronic (Bigal et al., 2006; Bigal and Lipton, 2006; Winter et al., 2012; Ornello et al., 2015). Moreover, there is evidence of a beneficial effect of weight loss on headache severity (Bond et al., 2011; Novack et al., 2011). In spite of these findings, literature data are far from being conclusive (Pakalnis and Kring, 2012; Ornello et al., 2015; Farello et al., 2017). Some authors did not confirm the association between overweight and migraine (Gilmore, 1999; Le et al., 2011; Pakalnis and Kring, 2012; Eidlitz-Markus et al., 2015). The disagreement may be due to the heterogeneity in the methodological designs and inclusion criteria of the patients. In adulthood, age and gender are considered as factors that may influence the relationship between weight and migraine (Ornello et al., 2015). A possible role of hormonal status in contributing to the migraine/body weight association has been proposed in women (Peterlin et al., 2010, 2013; Ornello et al., 2015). So far, only a few studies exploring the association between body weight and migraine severity in children and adolescents have been conducted (Pinhas-Hamiel et al., 2008; Kinik et al., 2010; Pakalnis and Kring, 2012; Ravid et al., 2013; Eidlitz-Markus et al., 2015). Moreover, some of them did not include the patients who referred to a specific pediatric headache center (Pinhas-Hamiel et al., 2008; Kinik et al., 2010; Ravid et al., 2013; Eidlitz-Markus et al., 2015).

Several elements support the hypothesis that the association between migraine and overweight may be multifactorial, involving both the central and peripheral pathophysiological mechanisms (Chai et al., 2014b; Oakley et al., 2014; Ravid, 2014; Farello et al., 2017). There is also an emerging evidence that some psychological factors, such as anxiety, depression, and stress, may influence this relationship (Ravid, 2014; Ornello et al., 2015). The association between headache and psychological symptoms has been widely documented. In particular, symptoms of anxiety and depression are very common among patients suffering from chronic migraine, both in adult (Antonaci et al., 2011; Kyungmi et al., 2014) and pediatric ages (Powers et al., 2006; Antonaci et al., 2011; Dindo et al., 2017).

Three main potential mechanisms have been hypothesized to explain this connection: (1) Stressful life events, anxiety, and depression may influence the onset, maintenance, and exacerbation of migraine (Merikangas et al., 1990; Radat and Swendsen, 2005; Antonaci et al., 2011); (2) pain itself may be a vulnerability factor leading to increased psychosocial problems, increased stress, anxiety, and depression (Merikangas et al., 1990; Radat and Swendsen, 2005; Antonaci et al., 2011); and (3) common determinants (e.g., neurotransmitters or receptors; Antonaci et al., 2011) and environmental, contextual, and interpersonal vulnerability factors can contribute to this association (Tarantino et al., 2017, 2018).

Little is known about the psychological profile of headache patients who suffer from being overweight. Data concerning the possible association between body weight, psychological factors, and headache are sparse, and involves only adult patients (Tietjen et al., 2007; Bond et al., 2015; Galioto et al., 2017). In adulthood, a significant interaction was found between some psychological symptoms, such as depression/anxiety, coping style, stress, migraine frequency, and overweight (Tietjen et al., 2007; Bond et al., 2015; Galioto et al., 2017).

### Purpose of the Present Study

Our study aimed at exploring the role of weight and psychological symptoms on migraine severity. In particular, we investigated: (1) the association between body weight and the frequency of migraine attacks, (2) the possible differences in anxiety and depression symptoms according to the frequency of attacks and body weight, and (3) the possible mediating role of anxiety and/or depression in the association between body weight and frequency of migraine attacks in children. We hypothesized that: (1) Overweight children would report a higher severity of pediatric migraine (i.e., frequency of attacks) compared to the normal weight ones; (2) anxiety and depression symptoms would be higher in children/adolescents complaining with a high frequency of attacks and in those who are overweight; and (3) anxiety and/or depression could mediate the relationship between being overweight and high frequency of headache.

## Materials and Methods

### Participants

This study was conducted prospectively from January 2015 to April 2017. Participants were recruited among children/adolescents who were referred for consultation to the Headache Center, Division of Neurology, of Bambino Gesù Children’s Hospital in Rome. One hundred twenty-four children/adolescents suffering from migraine without aura were involved. One hundred eleven of these were included in the study (47 boys and 64 girls; age range 8–18 years; mean age 11.7 ± 2.4 years). At the first visit, every patient underwent a neurological examination and a general physical exam including weight and height determination. Headache diagnosis was made according to the criteria of the International Classification of Headache Disorders, 3rd edition [ICHD-III; Headache Classification Committee of the International Headache Society (IHS), 2013]. We excluded from our study the following: (1) patients with the presence of any internal conditions or other neurological diseases (four participants); (2) children/adolescents who were taking drugs for migraine prophylaxis and other medications acting on the central nervous system (one patient); and (3) underweight patients (eight patients).

Children/adolescents who had previously received medications for acute attacks of pain (i.e., triptans, ibuprofen, and paracetamol) were not excluded from the study.

### Measures

#### Frequency of Migraine Attacks

Data on the course of migraine were issued from a headache diary given at the first consultation and brought back by the family at the second consultation. According to the aim of our study, we extrapolated only the frequency of the attacks. Patients were classified into two groups: (1) high frequency (HF; participants complaining from weekly to daily attacks) and (2) low frequency (LF; children/adolescents with ≤3 episodes per month). The aforesaid break point was chosen for three main reasons: (1) Children/adolescents suffering from intermediate and chronic frequency of attacks were too few to allow a reliable statistical analysis; (2) a mere distinction between episodic and chronic migraine would have led to pool patients with a high but not chronic frequency of attacks with patients with a very low frequency of attacks; and (3) patients in need of preventive treatment were separated from patients without such requirement (Tarantino et al., 2014).

#### Body Mass Index

The patients’ weight status was evaluated according to the body mass index (BMI), calculated as the body mass divided by the square of the body height. BMI was calculated taking into account the sex and age. According to BMI value, the patients of each group were classified as: (1) underweight (under 5th percentile), (2) normal weight (NW; from 5th to 85th), (3) overweight (from 85th to 95th), and (4) obese (over 95th; Cacciari et al., 2006). To overcome the unbalanced distribution of this variable in our sample (low number of obese subjects), the overweight and obese groups were merged together (OW; “overweight”).

#### Anxiety and Depressive Symptoms

The Italian Self Administrated Psychiatric Scales for Children and Adolescents battery of tests was the assessment tool used in our study (Cianchetti and Sannio Fascello, 2001). The battery of tests was structured in six scales, which can be used either together or separately; each scale is divided into several subscales. The SAFA assessment explores a wide series of psychiatric symptoms and psychological conditions: anxiety-related areas (SAFA-A), depression-related areas (SAFA-D), somatic concerns (SAFA-S), obsessive-compulsive symptoms (SAFA-O), psychogenic eating disorders (SAFA-P), and phobias (SAFA-F).

It leads to an individual profile (including one of the above-mentioned scales and the related subscales) and to a global profile. The entire battery of tests can be completed in 30–60 min.

In relation to age, the questionnaire was organized in two versions: “e” for patients aged 8–10 years and “m/s” for those aged 11–18 years. SAFA-A was divided in three forms: “e” for 8–10 years, “m” for 11–13 years, and “s” for 14–18 years. For each item, the subject has three possible answers: “true (scoring 2), partly true (scoring 1), and false (scoring 0).” The sum of points obtained in each scale and subscale can be converted into *T* scores, sten, points, and percentiles.

SAFA-A includes four subscales producing a “Total anxiety” score: “Generalized anxiety,” “Social anxiety,” “Separation anxiety,” and “School anxiety.” The questionnaire is composed of 42 items for the “e” version and 50 items for the “m/s.”

Also SAFA-D leads to a total score and includes seven subscales: “Depressed mood,” “Anhedony/disinterest,” “Toucy mood,” “Sense of inadequacy/low self-esteem,” “Insecurity,” “Feeling of guilt,” and “Hopelessness.” The scale consists of 48 items for the “e” version and 56 for the “m/s” version.

Within these scales, there are “items of lies,” six for SAFA-D “e,” and the same six plus one for SAFA-D “m-s,” in order to verify the reliability of the answers (Cianchetti and Sannio Fascello, 2001).

The SAFA battery was validated. It showed good psychometric properties (Cianchetti and Sannio Fascello, 2001; Franzoni et al., 2009; Pellicciari et al., 2012). In our study, the Cronbach’s test showed *α* values of 0.84 for answers to SAFA anxiety questions and of 0.77 for SAFA depression. Also, considering the SAFA Anxiety and Depression together, the *α* value was reliable (*α* = 0.81).

The same examiner (ST, with a specific training in psychological assessment in pediatric age) administered the psychological screening tests in a single session. The evaluation consisted of a psychological interview and the administration of the SAFA-A and SAFA-D questionnaires performed in the second visit. To avoid a possible effect of pain on the psychological evaluation, we made sure that all patients were free from headache 24 h before the psychological screening. Moreover, all patients had to be able to read, understand, and answer every item of the questionnaires.

#### Statistical Analysis

Statistical analysis was performed using the SPSS 22.0 software (Statistical Package for the Social Sciences). According to the aim of our study, the patients were grouped according to the frequency of attacks and weight.

Chi square test was used to verify the differences between NW and OW patients in terms of the frequency of attacks (high or low). The univariate logistic regression was used to estimate the risk of high-frequency of attacks associated with BMI.

The ANOVA test was used to compare the SAFA scores between the patients with low and high frequency of attack, and between NW and OW patients.

Statistical significance was determined using the two-tailed test and value of *p* was fixed at *p* < 0.05. A *post-hoc* correction for multiple comparisons was made using the Bonferroni’s test, setting the significance cut-off at a/n with *a* = 0.05 and final value of *p* = 0.025.

To verify the possible mediating effect of the anxiety and depressive symptoms on the frequency of the attacks, we carried out a mediation analysis using the Sobel’s test (*z*-value). In particular, the SAFA scores that proved significant in the ANOVA test were considered as possible mediators.

## Results

### Sample Characteristics

Fifty-five patients were normal weight (49.6%), while 56 patients (50.4%) were classified as overweight (overweight/obese). Overweight/obese patients had a mean BMI of 29.5 ± 5.2. Most children had a low frequency of attacks (60; 54.0%), whereas 51 patients (45.9%) reported a high frequency.

### Association Between Body Weight and the Frequency of Migraine Attacks

High frequency patients were more common in the OW group (64.7%) than among the NW patients (35.3%; OR 2,5; CI 1.1–6.3; *p* < 0.05). In contrast, 61.7% of the LF patients showed normal body weight, while 38.3% was overweight (Table 1). There were no gender differences between the HF and LF patients (60% females and 40% males vs. 53% females vs. 47% males, respectively; *χ*^2^ = 0.49; *p* > 0.05), and between the NW and OW children/adolescents (55% females and 45% males vs. 60% females and 40% males, respectively; *χ*^2^ = 0.32; *p* > 0.05). Also, the age was not different between the HF and LF patients (11.8 ± 2.4 years vs. 11.6 ± 2.1 years, *χ*^2^ = 0.44; respectively; *p* > 0.05), and between the NW and OW patients (11.6 ± 2.3 years vs. 11.1 ± 2.4 years, respectively; *χ*^2^ = 0.51; *p* > 0.05).

**Table 1:** Distribution of patients according to weight and frequency of attacks.

		Low frequency	High frequency	Total
Normal weight	Count	37	18	55
	% within weight	67.3%	32.7%	100.0%
	% within frequency	61.7%	35.3%	49.5%
Overweight	Count	23	33	56
	% within weight	41.1%	58.9%	100.0%
	% within frequency	38.3%	64.7%	50.5%
Total	Count	60	51	111
	% within weight	54.1%	45.9%	100.0%
	% within frequency	100.0%	100.0%	100.0%

### Difference in Anxiety and Depression Symptoms Between Children With High and Low Frequency of Migraine Attacks

High frequency children showed higher levels of total anxiety as compared with the LF patients (SAFA-A Tot: *F* = 15.107; *p* < 0.0001 (Table 2). In particular, with regard to the differences in the SAFA-Anxiety and Depression subscales between the groups, we found that the HF patients reported higher scores in all the SAFA-A subscales (*p* < 0.025), suggesting that the HF patients were more anxious in several fields, such as school (*p* < 0.025), social relationships (*p* < 0.025), and separation from parents (*p* < 0.025; Table 2). Although the HF patients showed a higher mean score in the SAFA-D Total scale, we did not find a significant difference between the two groups (SAFA-D Tot: *F* = 4.270; *p* < 0.025). No difference in the SAFA-D subscales was found between the HF and LF patients (*p* > 0.025; Table 2).

**Table 2:** Anxiety and depression symptoms in patients with high and low frequency of attacks.

	Low frequency	High frequency	Eta-squared	*p*
SAFA scales	Mean ± SD	Mean ± SD		
SAFA-A generalized anxiety	7.4 ± 4.7	11.3 ± 5.8	0.12	0.000^*^
SAFA-A social anxiety	5.9 ± 4.5	8.9 ± 5.3	0.09	0.001^*^
SAFA-A separation anxiety	6.2 ± 4.3	8.6 ± 4.8	0.06	0.006^*^
SAFA-A scholastic anxiety	7.0 ± 4.9	10.2 ± 5.6	0.08	0.002^*^
SAFA-A total anxiety	26.4 ± 15.1	38.2 ± 16.8	0.122	0.000^*^
SAFA-D depressed mood	3.1 ± 2.9	4.2 ± 3.9	0.02	0.133
SAFA-D anhedony	1.6 ± 1.7	2.4 ± 2.5	0.03	0.065
SAFA-D touchy mood	5.2 ± 3.4	6.6 ± 4.2	0.18	0.070
SAFA-D sense of inadequacy	2.7 ± 3.0	2.8 ± 3.1	0.07	0.943
SAFA-D insecurity	5.8 ± 3.6	7.2 ± 3.9	0.03	0.063
SAFA-D feeling of guilt	3.3 ± 2.8	4.2 ± 2.6	0.02	0.116
SAFA-D hopelessness	2.0 ± 2.5	2.4 ± 3.2	0.07	0.489
SAFA-D total depression	24.3 ± 15.2	31.7 ± 19.5	0.21	0.042

* Statistically significant differences.

### Difference in Symptoms of Anxiety and Depression Between Overweight and Normal Weight Children

Comparing the SAFA-Anxiety and Depression between the NW and OW patients, a significant difference between the groups was found. In particular, the OW patients showed significantly higher scores in the “Separation anxiety” subscale (*F* = 7.855; *p* < 0.01). No other differences between the groups were found in the SAFA-A subscales (SAFA-A Tot: *F* = 1.849; *p* > 0.025). We did not find differences between the NW and OW children/adolescents in the SAFA-D scales (SAFA-D Tot: *F* = 1.302; *p* > 0.025; Table 3).

**Table 3:** Anxiety and depression symptoms in normal and overweight patients.

	Normal weight	Overweight	Eta-squared	*p*
SAFA scales	Mean ± SD	Mean ± SD		
SAFA-A generalized anxiety	9.0 ± 5.6	9.4 ± 5.5	0.001	0.736
SAFA-A social anxiety	7.2 ± 5.0	7.4 ± 5.2	0.000	0.828
SAFA-A separation anxiety	6.0 ± 4.3	8.5 ± 4.8	0.067	0.006^*^
SAFA-A scholastic anxiety	8.1 ± 5.7	8.7 ± 5.3	0.003	0.551
SAFA-A total anxiety	29.6 ± 16.0	34.0 ± 17.7	0.017	0.177
SAFA-D depressed mood	3.4 ± 3.2	3.8 ± 3.6	0.005	0.494
SAFA-D anhedony	1.7 ± 1.8	2.4 ± 2.5	0.023	0.145
SAFA-D touchy mood	5.5 ± 3.5	6.3 ± 4.2	0.011	0.321
SAFA-D sense of inadequacy	2.7 ± 3.0	2.8 ± 3.1	0.001	0.798
SAFA-D insecurity	6.2 ± 3.7	6.7 ± 3.9	0.004	0.541
SAFA-D feeling of guilt	3.6 ± 2.5	3.8 ± 3.1	0.001	0.728
SAFA-D hopelessness	2.0 ± 2.6	2.4 ± 3.2	0.004	0.525
SAFA-D total depression	25.8 ± 15.4	30.0 ± 20.0	0.014	0.257

* Statistically significant differences.

### Anxiety and Depressive Symptoms as Mediators Between Body Weight and Frequency of Migraine Attacks

Sobel’s test showed a mediating role between being overweight and high frequency of attacks for social anxiety (*z* = 2.04 ± 0.03; *p* < 0.05) and total anxiety (*z* = 2.11 ± 0.03; *p* < 0.05). No mediation role was found for depression (*z* = 0.77 ± 0.02; *p* > 0.05), school anxiety (*z* = 1.86 ± 0.03; *p* > 0.05), and separation anxiety (*z* = 1.64 ± 0.02; *p* > 0.05).

## Discussion

Our study investigated the association between body weight, migraine frequency, and psychological symptoms. The main results were as follows: (1) Overweight status was associated with a high frequency of migraine attacks; (2) there was an association between the frequency of attacks, anxiety, and depression total scores; in particular, the HF patients showed a higher score in every SAFA-A subscale; (3) OW patients presented with higher symptoms of separation anxiety than NW patients; and (4) anxiety, in particular social anxiety, mediated the association between being overweight and the severity of migraine.

Over the last years, there has been an increasing interest in analyzing the association between headache and body weight, with controversial evidences (Le et al., 2011; Pakalnis and Kring, 2012; Verrotti et al., 2012; Winter et al., 2012; Chai et al., 2014b; Oakley et al., 2014; Ravid, 2014; Eidlitz-Markus et al., 2015).

The results of our study, showing an association between the overweight status and high frequency of migraine attacks, confirms the possible interaction between weight and migraine severity (Bigal et al., 2006; Bigal and Lipton, 2006; Winter et al., 2012; Oakley et al., 2014; Ornello et al., 2015). Several studies on adult populations showed that obesity and overweight may be involved in migraine progression. In particular, a high BMI and obesity lead to an increase in the frequency of attacks and progression from episodic to chronic migraine (Bigal et al., 2006; Bigal and Lipton, 2006; Pinhas-Hamiel et al., 2008; Kinik et al., 2010; Winter et al., 2012; Ravid et al., 2013; Ornello et al., 2015). Moreover, some studies suggested the role of BMI reduction in migraine outcome, both in the adult and pediatric age (Hershey et al., 2009; Bond et al., 2011; Novack et al., 2011; Verrotti et al., 2013). Interestingly, our results may have clinical implications for the management and treatment of pediatric migraine. Children and adolescents with recurrent headaches may require prophylactic therapy, which is usually prolonged for some months (Papetti et al., 2019). Most drugs currently used for migraine prophylaxis, such as amitriptyline and flunarizine, can lead to body weight increase (Young and Rozen, 2005; Silberstein, 2015). Since the BMI increase can worsen migraine, as confirmed by the present study, this factor should be taken into account, especially when the pharmacological treatment is associated with a paradoxical increase in the frequency of attacks. In these cases, the clinician should consider the use of other classes of drugs that do not interfere with body weight.

Among our patients, those suffering from a high frequency of attacks had a higher total anxiety, as compared to those with low frequency. In particular, the anxiety scores were higher in all SAFA-A subscales, showing that the children/adolescents with a high frequency of migraine attacks may experience more anxious feelings in several fields of life, such as social relationships, school, and separation from parents. Our results are in line with the previous evidence, describing children with migraine as shy, withdrawn, fearful of failure, and needing approval (Cunningham et al., 1987; Tarantino et al., 2017). Moreover, young migraineurs are more prone to anxiety symptoms and less able to cope with stress (Cunningham et al., 1987; Powers et al., 2006; Antonaci et al., 2011). Although the precise nature of the headache-anxiety-depression association is still unknown, the migraine course is probably influenced by these psychological symptoms (Merikangas et al., 1990; Guidetti et al., 1998; Antonaci et al., 2011).

To date, the psychological profile of overweight children and adolescents with migraine has not been investigated. In adults, Tietjen et al. (2007) showed that, among the patients seeking treatment for headache, anxiety, and depression were more common in obese patients than in non-obese ones. In our sample, the OW patients had higher symptoms of separation anxiety, as compared to the NW patients.

Previous studies showed the role of separation anxiety symptoms and attachment style on migraine clinical features in pediatric samples (Esposito et al., 2013; Tarantino et al., 2013, 2017; Faedda et al., 2018). There is also evidence that the attachment insecurity and mother-child relationship are involved in the emotional regulation and subsequent eating behavior (Strauss et al., 1985; Brødsgaard et al., 2014). In our study, the association between migraine, overweight, and separation anxiety symptoms may be due to the common psychological vulnerability factors. Migraine and obesity may share an increased risk for internalizing disorders, difficulties in emotional regulation, and dysfunctional mother-child interaction (Strauss et al., 1985; Strauss, 2000; Fichtel and Larsson, 2002; Antonaci et al., 2011; Brødsgaard et al., 2014; Tarantino et al., 2018). We hypothesize that overweight children with migraine may have low self-esteem and life satisfaction (Danielsen et al., 2012; Nazeri et al., 2018) so that they may feel more shy and hopeless and may be more prone to seek support from caregivers. From another perspective, the higher levels of separation anxiety symptoms in our *young overweight patients* may be related to a maternal maladaptive caring behavior. In a previous study, we found that the mothers of children suffering from migraine may show difficulties in understanding and detecting their own emotions and other people’s emotions (Tarantino et al., 2018). They may also appear less empathic to their children’s psychological needs and affectively less involved in the relationship with them (Tarantino et al., 2018). Moreover, we found an association between these maternal psychological factors (alexithymia) and anxiety symptoms, including separation anxiety, in their migraine children (Tarantino et al., 2018). From this point of view, in the present OW patients, the caregivers’ inability to decode and cope with their children’s needs, emotional requests, and pain signals may increase the children’s anxiety and lead mothers to use an inappropriate feeding behavior as a successful tool for calming or as a source of relationship (Evers et al., 2010; van Strien et al., 2013).

Very few studies explored the interaction between the psychological factors, body weight, and headache severity. Literature data showed that migraine and obesity share similar psychological features, such as anxiety, mood, and stress related disorders (Cunningham et al., 1987; Guidetti et al., 1998; Strauss, 2000; Fichtel and Larsson, 2002; Danielsen et al., 2012; Nazeri et al., 2018). Obese children and adolescents show low self-esteem, social anxiety, and social isolation (Strauss, 2000; Fichtel and Larsson, 2002; Danielsen et al., 2012). They may very often undergo stigmatization (Fichtel and Larsson, 2002). As discussed above, the same psychological characteristics have been described also in children with migraine (Cunningham et al., 1987; Masruha et al., 2012). In adult patients, a significant interaction was found between some psychological factors, such as depression/anxiety, coping style, stress, migraine frequency, and being overweight (Tietjen et al., 2007; Bond et al., 2015; Galioto et al., 2017). A previous study investigated the relationship of childhood headache with depression, anxiety, and eating habits but it did not consider the children’s weight status according to the BMI (Bektaş et al., 2015).

In the present study, mediation analysis showed that the anxiety symptoms, in particular social anxiety, may be a vulnerability factor influencing not only the frequency of migraine attacks but also the relationship between weight and migraine severity. Social anxiety may include emotional, behavioral, and physical symptoms (American Psychiatric Association, 2013). It is a condition characterized by an excessive and inappropriate worry about social situations, fear of being negatively evaluated, criticized, or rejected by others. Social anxiety is characterized by higher levels of distress and may interfere with daily routine, school, or other social activities (American Psychiatric Association, 2013). Previous literature data showed a high level of social anxiety in children suffering from migraine (Vulić-Prtorić et al., 2007). In particular, there is evidence that children with migraine, being more afraid of hurting themselves (Vulić-Prtorić et al., 2007), can more often get involved in solitary activities and are described as having a fewer peer relationship. We can hypothesize that, in our sample, social anxiety may be amplified by being overweight, causing in children the perception of feeling less “acceptable” and less able to meet social expectations. The difficult relationship with their own body weight would, therefore, influence their social relationships and self-confidence (Strauss, 2000; Fichtel and Larsson, 2002; Danielsen et al., 2012), increasing the feelings of hopelessness, isolation, and frustration. This can promote migraine onset, precipitate, and aggravate migraine attacks. The repetition of pain episodes may have a negative impact on the daily activity, as well as on school and social functioning, leading to the chronification of pain. Moreover, the repetition of pain episodes may increase the levels of stress (Merikangas et al., 1990), thus favoring a dysfunctional feeding behavior as a coping mechanism (van Strien et al., 2013; Nazeri et al., 2018).

These finding can provide important clinical consequences for an efficacious treatment program in children with migraine. Given the significant impact of both being overweight and anxiety on the migraine course, a special attention should be given to weight status and psychological symptoms. Detecting anxiety symptoms, in particular social anxiety, would mitigate the effect of weight on migraine severity and may reduce the risk of migraine chronification. A psychological intervention, such as cognitive-behavioral, may help children with migraine to identify and modify their negative thoughts about self, and may improve children’s self-esteem and coping ability. These may prevent the vicious cycle between weight, anxiety, and migraine, above mentioned.

Our study has some limitations. First, our sample included children/adolescents referred into our tertiary headache center, therefore, they may not be a representative of the pediatric migraine in the general population. Second, in future studies, it will be important to differentiate the overweight patients from the obese ones. Finally, the psychological tools (SAFA-A and D) used in our study to explore the anxiety and depression are self-report questionnaires. While they have proven to be valid instruments for screening, they are not diagnostic for anxiety and depression.

## Conclusion

This is the first study investigating the association between body weight and anxiety/depression in children suffering from migraine. We found that the anxiety symptoms, in particular social anxiety, may contribute to the association between being overweight and migraine severity. Because of the potential impact that the psychological symptoms and body weight can have on the migraine outcome, a systematic evaluation of children and adolescents with migraine should include both a psychological screening and a particular *concern about body weight*.

## Data Availability Statement

The datasets generated for this study are available on request to the corresponding author.

## Ethics Statement

The studies involving human participants were reviewed and approved by Bambino Gesù Ethics Committee. Written informed consent to participate in this study was provided by the participants’ legal guardian/next of kin.

## Author Contributions

ST, LP, and MV conceived and designed the study. ST and ADS collected the data. LP analyzed the data. MANF, VM, FU, and RM were involved in the data interpretation. LP, SG, and FV assisted in the preparation of the manuscript. ST and MV drafted the manuscript and were the main authors. All authors contributed to the article and approved the submitted version.

### Conflict of Interest

The authors declare that the research was conducted in the absence of any commercial or financial relationships that could be construed as a potential conflict of interest.

## Abbreviations

ICHD-III: Headache Classification Committee of the International Headache Society
HF: High frequency
LF: Low frequency
NW: Normal weight
OW: Overweight
SAFA: Psychiatric scales for self-administration for youths and adolescents
SAFA-A: SAFA-anxiety
SAFA-D: SAFA-depression
